# Brown Tumors Mimicking Skeletal Metastases: A Diagnostic Pitfall in Primary Hyperparathyroidism

**DOI:** 10.1002/ccr3.71543

**Published:** 2025-12-02

**Authors:** Uzma Akbar, Osama Ahmad, Fatima Sajjad, Khawar Saeed, Umer Dawar, Muhammad Salman Shah, Abdullah Afridi, Kamil Ahmad Kamil

**Affiliations:** ^1^ Khyber Teaching Hospital Peshawar Pakistan; ^2^ Khyber Medical College Peshawar Pakistan; ^3^ Internal Medicine Department Mirwais Regional Hospital Kandahar Afghanistan

**Keywords:** brown tumors, case report, osteolytic lesions, primary hyperparathyroidism

## Abstract

Brown tumors are rare but important manifestations of primary hyperparathyroidism (PHPT). These benign, fibrotic, and erosive bony lesions result from localized rapid osteoclastic activity driven by excessive parathyroid hormone levels. Although brown tumors are considered one of the most pathognomonic signs of PHPT, they are infrequently encountered in clinical practice. They closely mimic malignant osteolytic processes, posing a diagnostic challenge, especially in cases of undiagnosed hyperparathyroidism. Radiographic findings often resemble those of metastatic bone disease or primary bone malignancies.

## Key Clinical Message

This case underscores the importance of including primary hyperparathyroidism in the differential diagnosis for patients presenting with multiple osteolytic lesions, particularly when hypercalcemia is present. A structured approach involving biochemical assessment, imaging, and, if necessary, histological confirmation, can prevent unnecessary oncological investigations.

## 1 Background

Primary hyperparathyroidism (PHPT) is characterized by excessive secretion of parathyroid hormone (PTH), leading to hypercalcemia and various systemic manifestations. The classic mnemonic “psychic moans, abdominal groans, renal stones, and painful bones” summarizes the clinical features associated with hypercalcemia, including mood disturbances, gastrointestinal discomfort, nephrolithiasis, and bone pain [1]. Parathyroid adenomas account for approximately 85% of all cases of PHPT. It causes autonomous PTH secretion, disrupting calcium and phosphate homeostasis [2, 3]. Sustained elevation of PTH stimulates osteoclastic bone resorption, which may result in fibrotic and cystic lesions known as brown tumors (BTs). These benign lesions are characterized by fibroblastic proliferation, hemorrhage, and hemosiderin deposition, which give them their characteristic brown appearance [1, 4]. Despite being non‐neoplastic, BTs can mimic malignant or metastatic osteolytic lesions, posing significant diagnostic challenges [5, 6, 7], especially when the underlying hyperparathyroidism is undiagnosed. They typically affect the long bones, ribs, pelvis, and facial bones [1].

Although BTs are classically recognized as a typical skeletal issue associated with PHPT, they are infrequently observed in modern clinical settings. This infrequency, coupled with their radiological similarity to cancerous growths, can result in incorrect diagnoses and delayed treatment. Here, we present a case initially mistaken for skeletal metastases, later confirmed to be due to PHPT.

## 2 Case History

A 55‐year‐old female from Upper Dir presented to the Outpatient Department at Khyber Teaching Hospital, Peshawar, with chief complaints of weight loss and abdominal pain localized to the left hypochondrium for the past 3 months. The pain was described as dull, with an intensity of 7–8 on a 10‐point scale, and did not improve with any NSAIDs and opioids. She did not experience any additional symptoms, such as fever, vomiting, GERD, burning micturition, or constipation. Her medical history was negative for renal stones, trauma, fractures, or psychiatric issues.

### 2.1 Clinical Examination

During the physical examination, the only notable finding was tenderness just below the left costal margin. Additionally, a firm, non‐tender, irregular, and multinodular goiter was palpable.

### 2.2 Laboratory Investigations

Initial laboratory assessments indicated a hemoglobin level of 9.5 g/dL, mean corpuscular volume of 93 fL, mean corpuscular hemoglobin of 24 pg, total leukocyte count of 6.9 × 10^3^ cells/μL, and a platelet count of 128 × 10^3^ cells/μL. Inflammatory markers revealed a C‐reactive protein level of 2.7 mg/L and an erythrocyte sedimentation rate of 12 mm/h. The lactate dehydrogenase was 151 U/L, and the uric acid level was 4.8 mg/dL. Renal function tests and serum electrolyte levels were within normal limits. Alkaline phosphatase was significantly elevated at 2078 U/L, and serum calcium was 12.98 mg/dL. Thyroid function tests were normal, despite the presence of a multinodular goiter. The patient was treated with intravenous NSAIDs and opioids; however, her pain persisted.

### 2.3 Diagnostic Work Up

**FIGURE 1:**
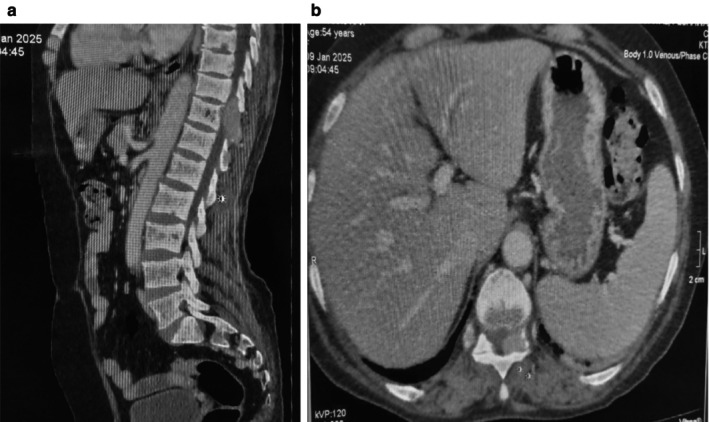
Sagittal and axial views of CT abdomen and pelvis showing osteolytic lesions at the D11/D12 level.

**FIGURE 2:**
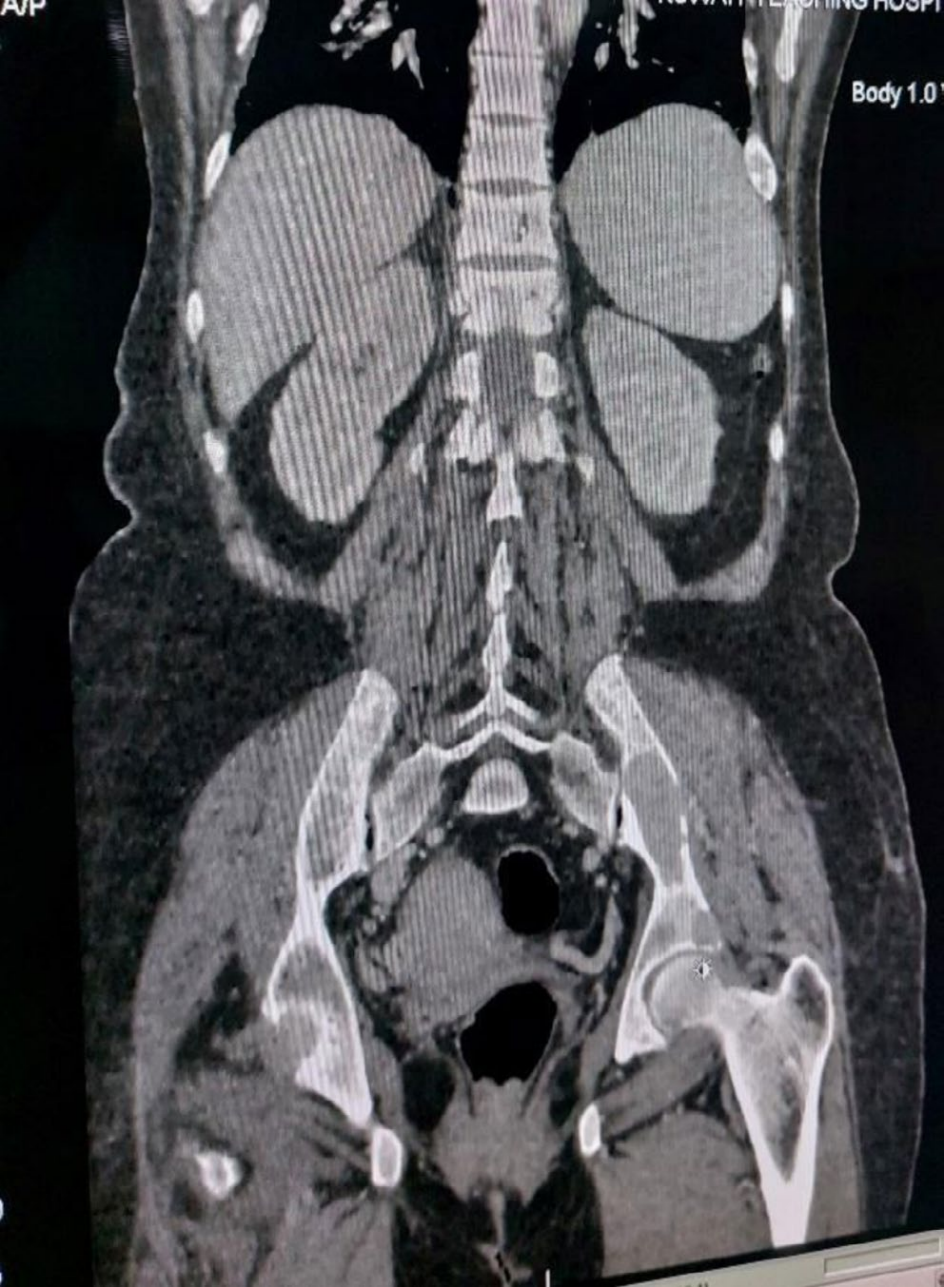
Coronal view of CT abdomen and pelvis showing hypodense lesions in the left iliac blade.

**FIGURE 3:**
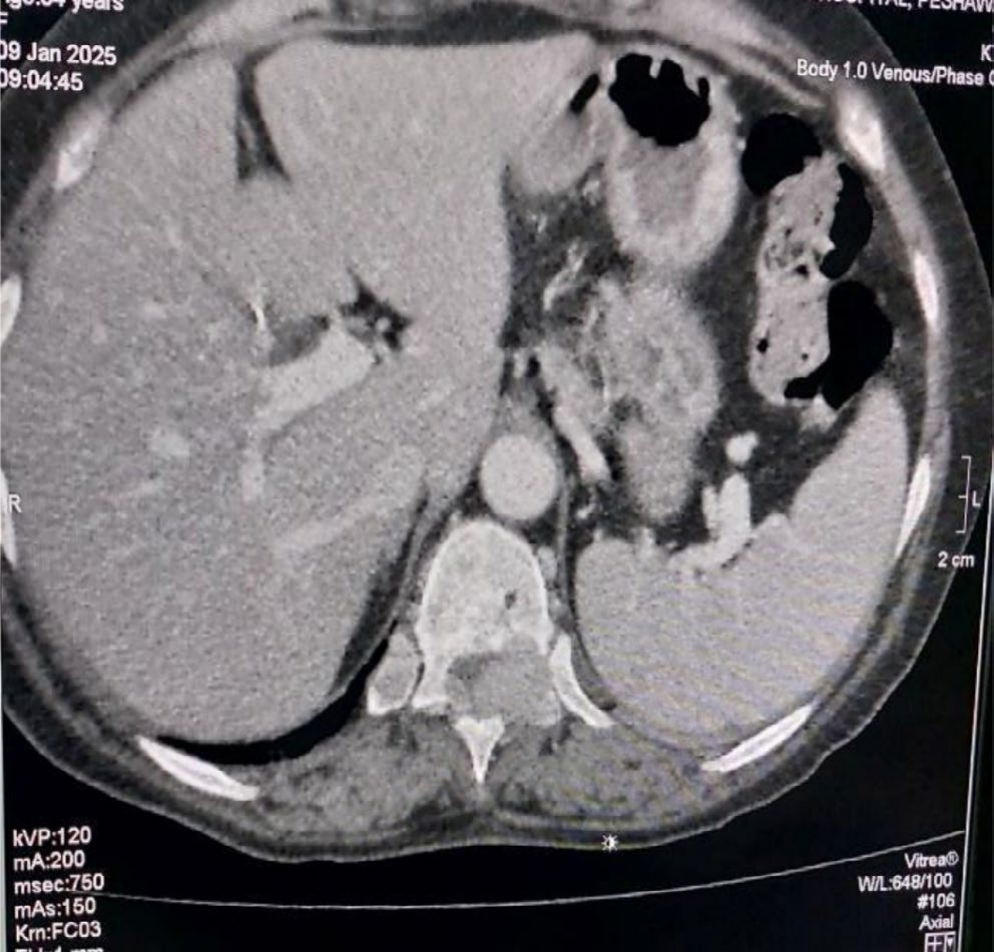
Axial view of CT abdomen and pelvis at the thoracic level showing an osteolytic lesion in one of the ribs.

Based on the available laboratory reports and patient symptoms, an initial suspicion of metastatic bone disease was considered. Consequently, further radiological investigations were conducted, including a CT chest, abdomen and pelvis. Imaging studies, including abdominal and pelvic ultrasonography and chest X‐ray (CXR), revealed no significant findings. However, neck ultrasound confirmed the presence of a multinodular goiter. Unexpectedly, CT‐TAP revealed lytic bone lesions in the D11/D12 vertebrae as shown in Figure 1a,b, left iliac blade (Figure 2), and multiple ribs (Figure 3), suggestive of metastatic deposits. A hypodense mass was also identified in the thyroid gland. Subsequently, a comprehensive diagnostic evaluation was performed to establish the diagnosis, including a bone scan to assess the distribution of osteolytic lesions. The scan confirmed the presence of lytic lesions at the same sites, that is, multiple ribs, the left iliac blade, and D11/D12 vertebrae.

Given these concerning findings, the primary suspicion was a thyroid malignancy with widespread bone metastasis; therefore, ultrasound‐guided fine‐needle aspiration cytology of the thyroid nodule was performed. The results classified it as Bethesda category II (i.e., benign cytology)/Thy 2 (benign nodular goiter), indicating that the thyroid lesion was incidental and unlikely to be the cause of the osteolytic lesions in the present case.

With thyroid malignancy ruled out, multiple myeloma was considered the next differential diagnosis. Investigations, including lateral skull radiography, bone marrow biopsy, and serum protein electrophoresis, were conducted, but the findings were inconsistent with this diagnosis. Given the unclear etiology of her symptoms, further evaluation for primary malignancies, including mammography and CA‐125 levels to rule out breast and ovarian malignancies, was performed, both of which were normal.

Considering the persistence of undetermined osteolytic lesions, an orthopedic consultation was sought, and a bone biopsy was advised to rule out the possibility of primary bone malignancy. This step was crucial, as histopathological differentiation between BTs and neoplastic lesions can be challenging in atypical presentations, as highlighted in multiple case studies [5, 6, 7]. Biopsy conducted at Shaukat Khanum Memorial Cancer Hospital & Research Centre indicated abundant giant cells in the background of spindle to epithelioid cells, suggestive of hyperparathyroidism. For confirmation, the slides were reviewed by independent pathology teams at the Aga Khan Laboratory and Shifa International Hospital, both of which reported consistent results, reinforcing the suspicion of hyperparathyroidism when correlated with the pathology reports.

Next, we assessed PTH and serum calcium levels. Ionized calcium was elevated at 6.05 mg/dL (reference range: 4.4–5.3 mg/dL), and intact PTH was markedly elevated at 1874 pg/mL (reference range: 15–68 pg/mL). These results confirmed the diagnosis of PHPT. The osteolytic lesions were therefore identified as BTs (osteitis fibrosa cystica)—a known skeletal manifestation of advanced hyperparathyroidism, which can closely mimic metastatic bone disease.

### 2.4 Localization and Management

**FIGURE 4:**
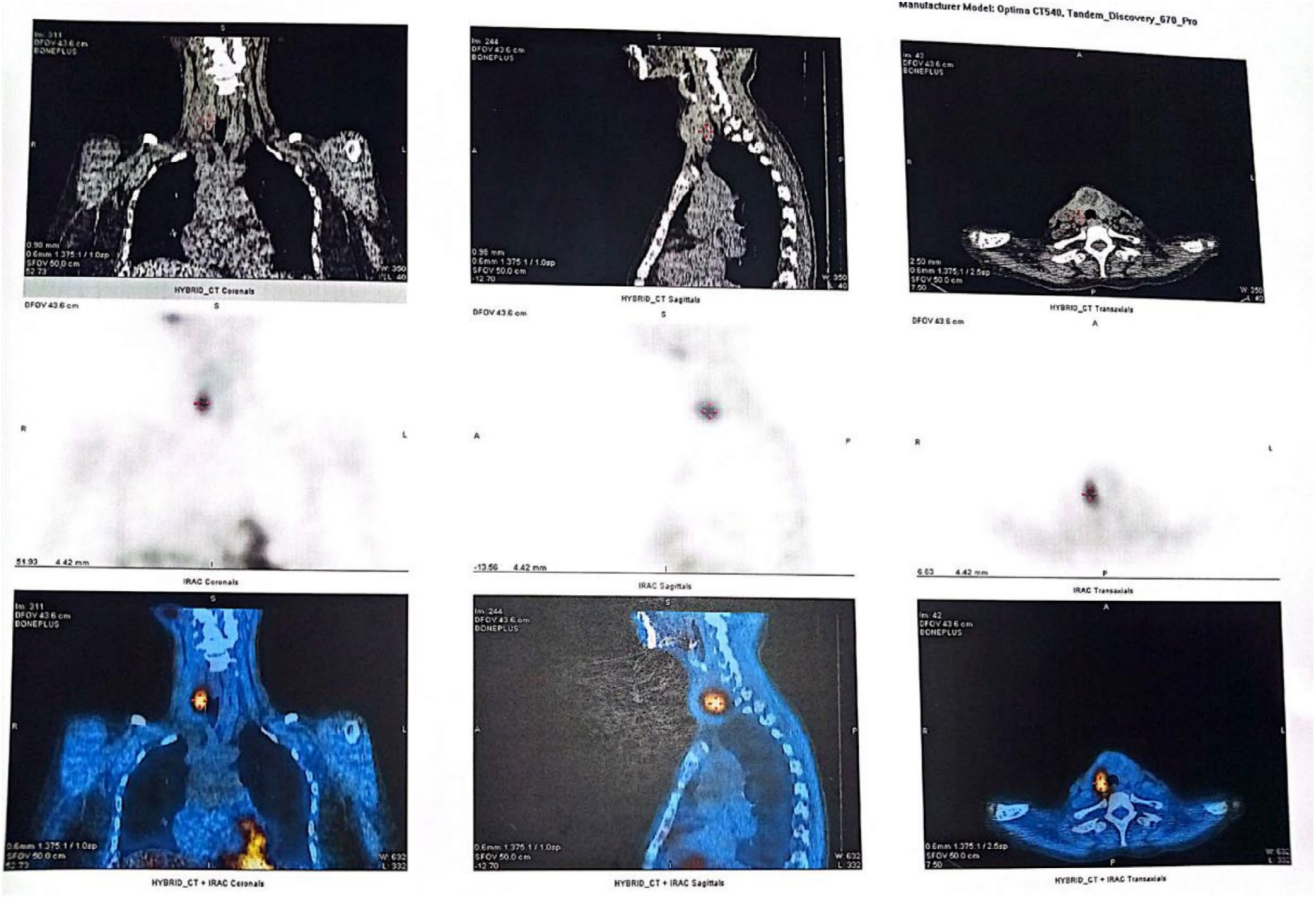
SPECT CT images showing a focal area of increased tracer uptake posterior to the enlarged right thyroid lobe. The thyroid gland appears enlarged and has a nodular appearance.

**FIGURE 5:**
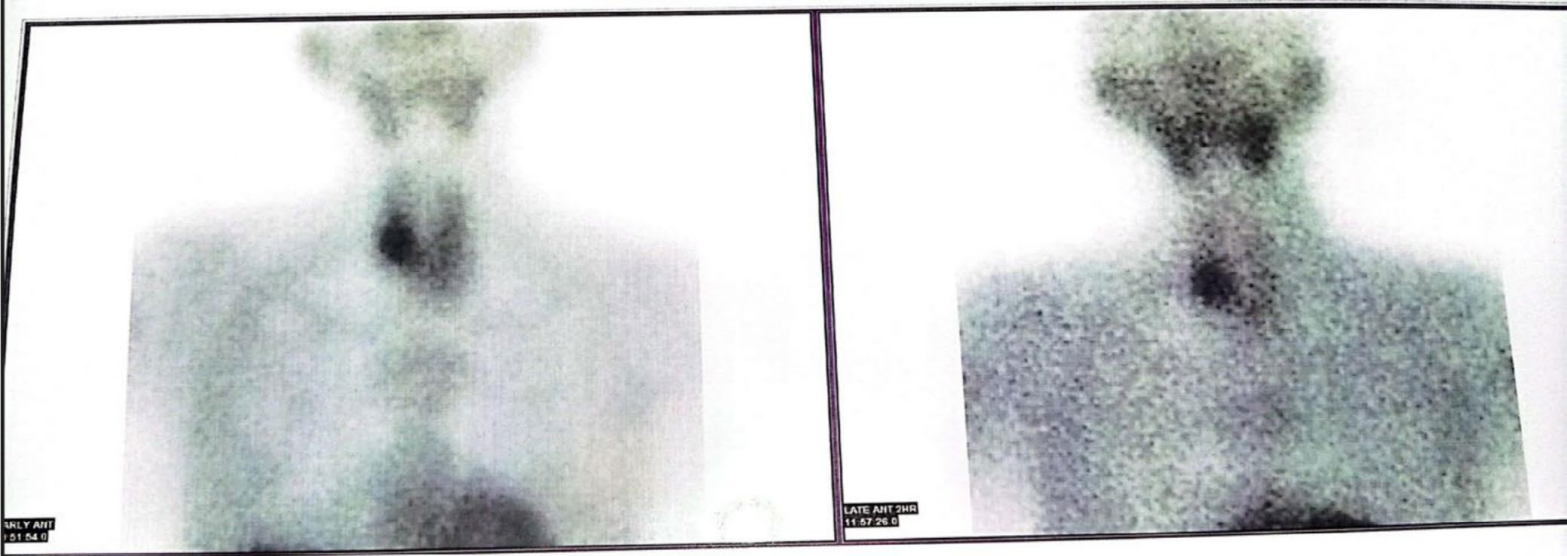
On the left, the early image shows a focal area of increased tracer uptake in the right thyroid lobe. The delayed image on the right shows persistence of the focal area of increased tracer uptake in the right thyroid lobe. The washout of the tracer was observed in the rest of the thyroid gland.

To localize the hyperfunctioning parathyroid gland, a sestamibi scan was performed. The scan revealed a parathyroid adenoma posterior to the enlarged right thyroid lobe in association with a multinodular goiter. Early images demonstrated focal tracer uptake in the region of the right thyroid lobe, and delayed images showed persistence of this uptake with washout from the remainder of the thyroid, consistent with parathyroid adenoma as shown in Figures 4 and 5.

Upon confirmation of the diagnosis, the patient was referred to the surgical department for definitive management, which involved surgical resection of the parathyroid adenoma.

### 2.5 Follow‐Up and Outcome

The patient underwent parathyroidectomy and reported complete resolution of pain at the subsequent follow‐up visit. For ongoing management, biochemical monitoring with serum calcium, PTH, and alkaline phosphatase was scheduled at 3–6 month intervals to ensure metabolic stability and assess skeletal recovery.

## 3 Discussion

Primary hyperparathyroidism is an endocrine disorder characterized by the overproduction and release of PTH from one or more of the four parathyroid glands. The primary etiology of PHPT is parathyroid adenoma, which is responsible for approximately 80% of cases, followed by parathyroid gland hyperplasia (5%–20%), while parathyroid carcinoma accounts for < 1% [1, 8]. Patients with PHPT often present with symptoms of hypercalcemia, such as bone pain, fractures, nephrolithiasis, abdominal discomfort, psychic manifestations, and, in severe cases, cardiac arrhythmias and coma [8].

Brown tumors, also known as osteoclastomas or osteitis fibrosa cystica, are noncancerous bone lesions that arise from long‐standing hyperparathyroidism [2]. They represent a rare clinical manifestation, occurring in approximately 3% of patients with PHPT and 2% of those with secondary hyperparathyroidism [7, 9]. Grossly, they appear as smooth, brown‐colored masses that may contain cystic areas. On a microscopic level, they are characterized by localized regions where the marrow is replaced by fibrous tissue with blood vessels and giant cells resembling osteoclasts, along with bleeding and hemosiderin deposits. Histologically, distinguishing a BT from a reparative granuloma is challenging because of their similar appearance (multinucleated macrophages and reactive fibrous tissue growth), necessitating pathological evaluation to rule out malignancy. Identifying evidence of hyperparathyroidism favors BTs over all other possible differentials [9].

The clinical presentation of BTs is diverse and nonspecific, including weakness, weight loss, bone pain, pathological fractures, and progressive bone swelling [5]. They typically affect long bones, ribs, pelvis, and facial bones [1]; although involvement of the mandible is very common, it was not observed in this case.

Our patient primarily complained of left‐sided chest pain for 3 months, unrelieved by analgesics, and a history of weight loss. Symptoms associated with hypercalcemia such as paresthesia, headaches, recent fractures, constipation, polyuria, and polydipsia may also be present, although many patients are asymptomatic and diagnosed incidentally [1, 2].

In addition to PHTP, the key differential diagnoses for osteolytic bone lesions include bone metastases, giant cell tumors (GCTs), multiple myeloma, and sarcomas. Other possibilities include tonsillar cysts, chondromas, and aneurysmal bone cysts [5, 7]. Diagnostic uncertainty commonly arises when multiple lytic lesions are identified across different skeletal sites, mimicking metastatic diseases or primary bone tumors [5]. Despite the classic association of hyperparathyroidism with hypercalcemia, the initial suspicion leaned toward malignancy due to the extensive bone involvement. Approximately 90% of patients with skeletal metastases present with multiple lesions [10]. Several studies [2, 4, 5, 7] have reported PHPT cases that were initially misdiagnosed as metastatic bone disease or primary bone tumors due to multiple osteolytic lesions. Similarly, in this case, the extensive lytic lesions involving multiple ribs, thoracic vertebrae, and the iliac bone led to a strong suspicion of metastatic disease.

For example, a case reported by Ming‐Chun Hsieh et al. described a 32‐year‐old woman who suffered a minor fall and subsequently developed severe pain in the left distal thigh, hip, and right shoulder. Imaging revealed marked osteopenia, multiple osteolytic bone lesions, and a pathological fracture of the left femur. Initially suspected to be metastatic disease, surgical intervention was undertaken; however, pathological findings confirmed PHPT with osteitis fibrosa cystica [11].

Distinguishing BTs from metastatic lesions, GCTs, or multiple myeloma is critical but challenging, as all may present with skeletal pain, hypercalcemia, osteolytic lesions on imaging, and multinucleated giant cells histologically [12, 13]. Panagopoulos et al. [7] reported a case of a 53‐year‐old man initially diagnosed with a GCT based on osteolytic lesions in the distal ulna and radius, but subsequent evaluation revealed underlying PHPT with BTs. Similar misdiagnosed cases have also been reported by Pezzillo et al., Jouan et al., and Mansfield et al. [14, 15, 16].

BT and GCT share overlapping clinical and radiological features, making differential diagnosis challenging. Histologically, both exhibit a highly vascular fibrotic stroma with multinucleated giant cells, complicating diagnosis based solely on bone specimens. Therefore, it is essential for pathologists to have access to comprehensive clinical and laboratory data, especially serum calcium and PTH levels, to guide diagnosis. In rare instances, concomitant GCTs and BTs have been reported. GCT should be suspected if bone lesions fail to regress following successful treatment of hyperparathyroidism [1, 7].

Multiple myeloma was another important differential diagnosis for this patient, given its high prevalence of bone involvement at the time of diagnosis (up to 80% of cases) [17]. However, the absence of the M band on serum protein electrophoresis and the lack of Bence‐Jones proteins in the urine suggested an alternative diagnosis. Similar diagnostic dilemmas have been reported by Wani et al. [10] and Chuang et al. [18].

In the diagnostic evaluation of suspected hyperparathyroidism with skeletal involvement, cervical ultrasound and technetium 99m sestamibi scintigraphy are key imaging modalities [1, 5]. Sestamibi scan reveals radiotracer uptake in both thyroid and parathyroid tissue during early phases; however, thyroid uptake diminishes over time, whereas parathyroid adenomas retain and intensify uptake over approximately 2 h. This persistent activity distinguishes parathyroid lesions from surrounding thyroid tissue [1, 5].

The primary treatment of BTs secondary to PHPT is resection of the hyperfunctioning parathyroid gland, as was advised in this case. Parathyroidectomy typically causes significant clinical improvement, normalization of biochemical parameters, and recovery of bone mineral density, with a low complication rate [1, 5, 8]. Parathyroid adenoma, as in this case, is a common cause of PHPT and its resection is curative [5, 7]. Christiansen et al. observed that bone changes typically start to regress within 6 months after parathyroidectomy in patients with PHPT. This regression was accompanied by a decline in bone turnover markers, underscoring the value of monitoring serum calcium, PTH, and bone markers, along with periodic bone density assessment, during follow‐up [19].

PHPT must be considered when evaluating patients with osteolytic bone lesions, even when the clinical presentation is atypical. Although metastatic disease, primary bone tumors, and multiple myeloma are common initial considerations, PHPT can present similarly with extensive bone involvement. A comprehensive diagnostic approach, including serum calcium and PTH levels, appropriate imaging, and histopathological analysis, is vital for ensuring accurate diagnosis and timely management, ultimately improving patient outcomes.

## 4 Conclusion

This case report highlights the importance of considering BTs in the differential diagnosis of multifocal osteolytic bone lesions. Early detection and treatment of conditions such as hyperparathyroidism can prevent further complications and improve patient outcomes. This case also emphasizes that laboratory tests for serum phosphate, calcium, and PTH levels should always be part of the routine evaluation of patients with osteolytic bone lesions. Routine calcium and PTH testing should be part of the workup for patients with unexplained lytic lesions, especially in the absence of confirmed malignancy.

## Author Contributions

**Uzma Akbar:** writing – original draft. **Osama Ahmad:** writing – review and editing. **Fatima Sajjad:** conceptualization. **Khawar Saeed:** visualization. **Umer Dawar:** methodology. **Muhammad Salman Shah:** resources. **Abdullah Afridi:** project administration. **Kamil Ahmad Kamil:** supervision.

## Funding

The authors have nothing to report.

## Consent

The authors certify that they have obtained all appropriate written patient consent forms. In the form the patient has given his consent for his images and other clinical information to be reported in the journal. The patient understands that his name and initials will not be published, and due efforts will be made to conceal his identity.

## Conflicts of Interest

The authors declare no conflicts of interest.

## Data Availability

The data that support the findings of this study are available on request from the corresponding author. The data are not publicly available due to privacy or ethical restrictions.

## References

[1] A. M. Alrotoie , A. A. Aljohani , R. Alrehaili , M. Alharbi , and Y. M. Alalawi , “Osteolytic Lesions (Brown Tumors) of Primary Hyperparathyroidism: A Report of Two Cases,” Cureus 16, no. 6 (2024): e61708. 38975429 10.7759/cureus.61708 PMC11225032

[2] A. D. Shetty , J. Namitha , and L. James , “Brown Tumor of Mandible in Association With Primary Hyperparathyroidism: A Case Report,” Journal of International Oral Health 7 (2015): 50–52. 25859108 PMC4377151

[3] S. J. Silverberg , B. L. Clarke , M. Peacock , et al., “Current Issues in the Presentation of Asymptomatic Primary Hyperparathyroidism: Proceedings of the Fourth International Workshop,” Journal of Clinical Endocrinology & Metabolism 99, no. 10 (2014): 3580–3594, 10.1210/jc.2014-1415. 25162667 PMC5393491

[4] I. B. Staouni , M. Haloua , B. Nizar , et al., “Primary Hyperparathyroidism Presenting as a Brown Tumor in the Mandible: A Case Report,” Radiology Case Reports 17, no. 6 (2022): 2283–2286. 35574569 10.1016/j.radcr.2022.03.081 PMC9092295

[5] C. Ayadi , S. Lanjery , H. Andour , et al., “Multiple Brown Tumors in Primary Hyperparathyroidism,” Radiology Case Reports 17, no. 11 (2022): 4239–4243. 36120515 10.1016/j.radcr.2022.07.110 PMC9471336

[6] V. G. Schweitzer , N. W. Thompson , and K. D. McClatchey , “Sphenoid Sinus Brown Tumor, Hypercalcemia, and Blindness: An Unusual Presentation of Primary Hyperparathyroidism,” Head & Neck Surgery 8, no. 5 (1986): 379–386, 10.1002/hed.2890080509. 3793483

[7] A. Panagopoulos , I. Tatani , H. P. Kourea , Z. T. Kokkalis , K. Panagopoulos , and P. Megas , “Osteolytic Lesions [Brown Tumors] of Primary Hyperparathyroidism Misdiagnosed as Multifocal Giant Cell Tumor of the Distal Ulna and Radius: A Case Report,” Journal of Medical Case Reports 12, no. 1 (2018): 176. 29936913 10.1186/s13256-018-1723-y PMC6016128

[8] A. Fazaa , Y. Makhlouf , S. Miladi , et al., “Hyperparathyroidism: Unusual Location of Brown Tumors,” Clinical Case Reports 10 (2022): e05376, 10.1002/ccr3.5376. 35140968 PMC8813670

[9] V. Grulois , I. Buysschaert , J. Schoenaers , F. Debruyne , P. Delaere , and V. Vander Poorten , “Brown Tumour: Presenting Symptom of Primary Hyperparathyroidism,” B‐ENT 1 (2005): 191–195. 16429752

[10] M. M. Wani , M. A. Halwai , B. A. Mir , A. Hussain , and N. Akhter , “Primary Hyperparathyroidism Masquerading Malignancy: A Case Report,” Journal of Medical Cases 1, no. 1 (2010): 27–28, 10.4021/jmc2010.07.106e.

[11] M. C. Hsieh , J. Y. Ko , and H. L. Eng , “Pathologic Fracture of the Distal Femur in Osteitis Fibrosa Cystica Simulating Metastatic Disease,” Archives of Orthopaedic and Trauma Surgery 124, no. 7 (2004): 498–501. 15168133 10.1007/s00402-004-0697-y

[12] L. Tram , M. Kubik , K. Kvist Jensen , and C. E. Almasi , “Brown Tumor Mimicking Metastases—The Late Manifestation of Hyperparathyroidism,” Acta Radiologica Open 11, no. 9 (2022): 20584601221128415, 10.1177/20584601221128415. 36132124 PMC9484045

[13] A. A. Khan , D. A. Hanley , R. Rizzoli , et al., “Primary Hyperparathyroidism: Review and Recommendations on Evaluation, Diagnosis, and Management. A Canadian and International Consensus,” Osteoporosis International 28 (2017): 1–19. 10.1007/s00198-016-3716-2 PMC5206263 27613721

[14] F. Pezzillo , R. Di Matteo , F. Liuzza , et al., “Isolated Bone Lesion Secondary to Hyperparathyroidism: Diagnostic Considerations,” Clinical Therapeutics 159, no. 4 (2008): 265–268. 18776985

[15] A. Jouan , L. Zabraniecki , V. Vincent , E. Poix , and B. Fournié , “An Unusual Presentation of Primary Hyperparathyroidism: Severe Hypercalcemia and Multiple Brown Tumors,” Joint Bone Spine 75, no. 2 (2008): 209–211. 18222720 10.1016/j.jbspin.2007.03.004

[16] L. Vera , M. Dolcino , M. Mora , et al., “Primary Hyperparathyroidism Diagnosed After Surgical Ablation of a Costal Mass Mistaken for Giant‐Cell Bone Tumor: A Case Report,” Journal of Medical Case Reports 5 (2011): 596. 22204520 10.1186/1752-1947-5-596 PMC3261225

[17] B. S. Mansfield and F. J. Raal , “Malignant Mimic: Brown Tumours of Primary Hyperparathyroidism,” Journal of Clinical & Translational Endocrinology: Case Reports 25 (2022): 100125, 10.1016/j.jecr.2022.100125.

[18] T. C. Chuang , J. M. Chang , S. J. Hwang , P. J. Hsiao , and Y. H. Lai , “A Patient of Primary Hyperparathyroidism With Full‐Blown Bone Changes Simulating Malignancy,” Kaohsiung Journal of Medical Sciences 14, no. 9 (1998): 584–589. 9796203

[19] P. Christiansen , T. Steiniche , K. Brixen , et al., “Primary Hyperparathyroidism: Short‐Term Changes in Bone Remodeling and Bone Mineral Density Following Parathyroidectomy,” Bone 25, no. 2 (1999): 237–244, 10.1016/S8756-3282(99)00162-1. 10456391

